# Prognostic value of IKBIP in papillary renal cell carcinoma

**DOI:** 10.1186/s12894-023-01290-x

**Published:** 2023-07-15

**Authors:** Huiling Zhang, Rui Tang, Xue Wen, Jingbo Cai, Juan Huang, Li Luo, Zhihui Yang

**Affiliations:** Department of Pathology, The Affiliated Hospital of Southwest Medical University, Luzhou, 646000 People’s Republic of China

**Keywords:** IKBIP, Papillary renal cell carcinoma, Signaling pathway, Prognosis, Bioinformatics

## Abstract

**Background:**

I kappa B kinase interacting protein, a highly conserved gene, has rarely been reported in cancer. According to previous study, IKBIP has only been shown to promote malignant progression of glioma. In other malignant tumors, few reports have examined the function of IKBIP, especially in papillary renal cell carcinoma. Therefore, the molecular profiles and clinical prognostic values of the IKBIP in papillary renal cell carcinoma remain undetermined.

**Methods:**

Several bioinformatic platforms and Immunohistochemistry were used to clarify the expression and prognostic values of IKBIP in Papillary renal cell carcinoma.

**Results:**

In this study, GEPIA and TIMER platform were used to identify mRNA expression of IKBIP in papillary renal cell carcinoma. And our results revealed that IKBIP mRNA expression was up-regulated in papillary renal cell carcinoma than in its corresponding normal tissues. In addition, high mRNA expression levels of IKBIP were correlated with age, pathological stage, pathological T stage and pathological N stage. Moreover, High IKBIP mRNA expression was negatively correlated with overall survival (OS) and disease-free survival (DFS) in patients of papillary renal cell carcinoma. Besides, Multivariate analysis indicated that IKBIP mRNA expression was an independent prognostic factor for patients of papillary renal cell carcinoma. Furthermore, Kyoto Encyclopedia of Genes and Genomes (KEGG) pathway analysis showed IKBIP co-expressed genes were enriched in homologous recombination, DNA replication, cell cycle, Mismatch repair, Fanconi anemia pathway, P53 signaling pathway and nucleotide excision repair. And Immunohistochemical profile showed that protein expression of IKBIP was higher in papillary renal cell carcinoma than adjacent normal tissue.

**Conclusions:**

Overall, our findings reveled that IKBIP may act as a novel and potential tumor factor to accelerate papillary renal cell carcinoma progression, meanwhile, IKBIP could serve as a promising target for treating papillary renal cell carcinoma.

## Introduction

Renal cell carcinoma (RCC) is the 13^th^ most common malignancy globally [1], accounting for 2–3% of all adult malignancies [2]. It is divided into three major histologic subtypes: clear cell RCC (ccRCC), papillary cell RCC (PRCC), and chromophobe RCC (ChRCC). PRCC is the second most common RCC subtype following clear cell RCC and occurring in up to 13–20% of renal epithelial tumors. It was defined as a malignant renal cell carcinoma with characteristic papillary structure, cytogenetic feature and specific Immunohistochemical profile [3–5] Most PRCCs occur in adults, but occurrence in childhood is possible. In recent years, there has been a little knowledge regarding the genetic profile of PRCC, and therapeutic advances of it were less studied. Therefore, it is important for us to explore the novel sensitive and potential biomarkers of PRCC.

I kappa B kinase interacting protein (IKBIP), also termed as IKIP, located within 0.5 kb upstream of APAF1(apoptotic protease-activating factor 1) on human chromosome 12. According to previous reports, APAF1 plays an important role in apoptosis and p53 has been shown to induce the expression of APAF1 and IKBIP at the transcriptional level [6]. The close linkage between APAF1 and IKBIP suggested that they may share the common regulation and biological functions. IKBIP is a p53 target gene with proapoptotic function and the expression of IKBIP depends on P53 [7]. But in glioma, IKBIP was reported to be up-regulated, the result indicated that IKBIP might have a dualistic nature in gliomagenesis [8]. Furthermore, IKIBP interacts with IKKα/β and acts as a negative regulator of NF-kB signaling, thus inhibiting NF-kB activation and proinflammatory cytokine production [9]. Function of IKBIP in malignant tumors has been rarely reported in previous studies. Only several articles reported that IKBIP was associated with more aggressive phenotypes of gliomas [10]. So far, Few reports have examined the role of IKBIP in PRCC. And this research has mainly focused on the correlation between IKBIP with clinicopathological characteristics and prognosis in PRCC patients.

Here, we compared the IKBIP mRNA expression between PRCC tissues and normal tissues in The Cancer Genome Atlas (TCGA). And the protein expression of IKBIP in normal tissues and PRCC was detected respectively by Immunohistochemistry. Furthermore, we evaluated the correlation between clinical characteristics and IKBIP mRNA expression of PRCC patients. Meanwhile, we explored prognostic value of IKBIP in PRCC. And with GO annotation and KEGG pathway analysis, we investigated the biological process and signaling pathways that related to IKBIP. Our results revealed that IKBIP was a new and promising prognostic biomarker for PRCC.

## Materials and methods

### Gene expression

Tumor Immune Estimation Resource (TIMER) platform (https://cistrome.shinyapps.io/timer/) and The Gene Expression Profiling Interactive Analysis (GEPIA) database (http://gepia.cancer-pku.cn/) were used to evaluate IKBIP mRNA expression in Pan-cancer based on RNA sequencing datasets from TCGA project. The xiantaoplatform (http://www.xiantao.love), which was built based on R, was used to conduct a comparison of IKBIP expression in PRCC with paired benign tissues and normal tissues respectively, Moreover, the protein expression of IKBIP in PRCC was detected by Human Protein Atlas (HPA) database (https://www.proteinatlas.org/).

### Clinical characteristics correlation

UALCAN (http://ualcan.path.uab.edu/index.html) database was used for the analysis of relative expression of IKBIP across tumor and normal cases, as well as in diverse tumor sub-groups. The normalized RNA sequencing data and corresponding clinicopathological details, which contained 290 PRCC cases and 32 normal renal tissue cases, were obtained from TCGA-KIRP database (https://portal.gdc.cancer.gov/). And the xiantaoplatform (http://www.xiantao.love) was used to evaluate the correlation between clinical characteristics and IKBIP mRNA expression of PRCC patients from TCGA project.

### Prognostic value in PRCC

Kaplan–meier Plotter (https://kmplot.com/analysis/) was used to analyze the prognostic value, including overall survival and disease-free survival, of IKBIP expression in PRCC based on TCGA expression data.

### Gene expression correlation and enrichment analysis

We evaluated the correlations of IKBIP expression with all human genes by The LinkedOmics Database Analysis (http://linkedomics.org/login.php). IKBIP co-expression was calculated statistically by using pearson’s correlation coefficient. The enrichment analysis of IKBIP co-expression, such as biological process and related signaling pathways, was performed by the gene set enrichment analysis (GSEA).

### Immunohistochemical analysis

We collected 15 pairs of formalin-fixed, paraffin-embedded (FFPE) specimens of PRCC and corresponding normal tissues from department of pathology in The Affiliated Hospital of Southwest Medical University(Luzhou, China) (KY2022310) between January 1^st^ 2014 and May 15^th^ 2022. All patients didn’t receive radiotherapy and chemotherapy before surgery. The ethics of this study has been approved by the Clinical Trial Ethics Committee of Affiliated Hospital of Southwest Medical University.

Immunohistochemical staining was performed using the Envision method, and the operation steps were carried out strictly according to the product instructions. Primary antibody IKBIP (HPA038677, dilution 1:100) was purchased from atlas antibodies. The Secondary antibody and DAB were purchased from Dako company. PBS was used as a negative control.

IKBIP localizes to endoplasmic reticulum (ER) [6], So we consider cytoplasmic staining as IKBIP positive expression. According to Histochemistry score (H-score), the intensity of IKBIP expression was scored 0,1,2 and 3 based on the intensity of staining, the percentage of positive cells was scored 0,1,2 and 3 based on ≤ 25%, 26%-50%, 51%-75%, ≥ 76%. The total H-score was calculated multiplying the two scores. And the H-score ranges from 0 to 9. H-score ≤ 4 was defined as low IKBIP expression, H -score > 4 was defined as high IKBIP expression. The results were evaluated by three clinical pathologists through double-blind method independently and final score was unified by them.

### Statistical analysis

We compared relative mRNA expression of IKBIP across PRCC and normal tissue cases by using Mann–Whitney U test. And chi-square test and Fisher’s precision probability test were utilized for the analysis of correlations between IKBIP mRNA expressions and clinicopathological characteristics. Meanwhile, we compared IKBIP protein expression across PRCC and paired tissues by using Fisher’s precision probability test. Kaplan–Meier curves and Multivariate cox analysis were used to explore the prognostic value of IKBIP in PRCC. *P* < 0.05 was considered statistically significant.

## Results

### IKBIP mRNA expression is significantly increased in human papillary renal cell carcinoma

**Fig. 1:**
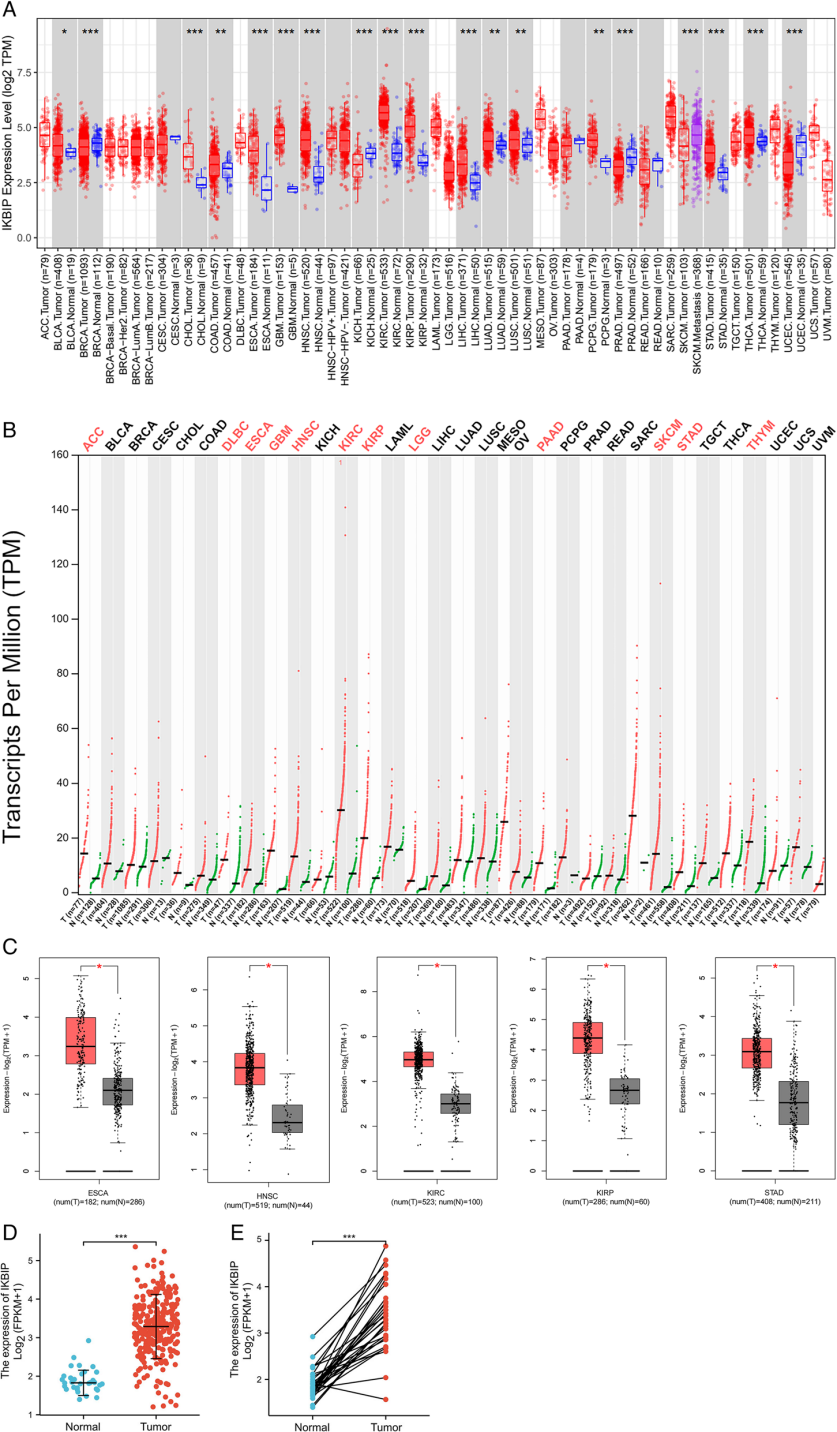
The expression levels of IKBIP in multiple cancer types were analyzed by several bioinformatics platforms based on TCGA database. **A** and **C** The mRNA expression profiles of IKBIP in multiple cancer types from GEPIA databases. **B** The mRNA expression profiles of IKBIP in multiple cancer types from TIMER databases. **D** IKBIP mRNA expression in PRCC tissues and normal tissues. **E** IKBIP mRNA expression in PRCC tissues and adjacent tissues. (*, *P* < 0.05; **, *P* < 0.01; ***, *P* < 0.001)

We analyzed IKBIP mRNA expression in multiple cancer types by using publicly assessable datasets from two bioinformatic platforms, including GEPIA and TIMER database. And we discovered that IKBIP mRNA expression showed an obvious increase in papillary renal cell carcinoma, esophageal squamous cell carcinoma, head and neck squamous cell carcinoma, renal clear cell carcinoma and gastric cancer compared with adjacent tissues and corresponding normal tissues (Fig. 1A-C). Based on TCGA-KIRP data, IKBIP mRNA expression was found to be higher in 289 PRCC samples than in 32 normal tissues through unpaired sample test (*P* < 0.001) (Fig. 1D). And IKBIP up-regulation was also confirmed in pairwise comparison of 32 PRCC tissues with matched adjacent benign tissues derived from the TCGA project (*P* < 0.001) (Fig. 1E).

### Correlation between IKBIP mRNA expression and clinicopathological features of PRCC patients

**Fig. 2:**
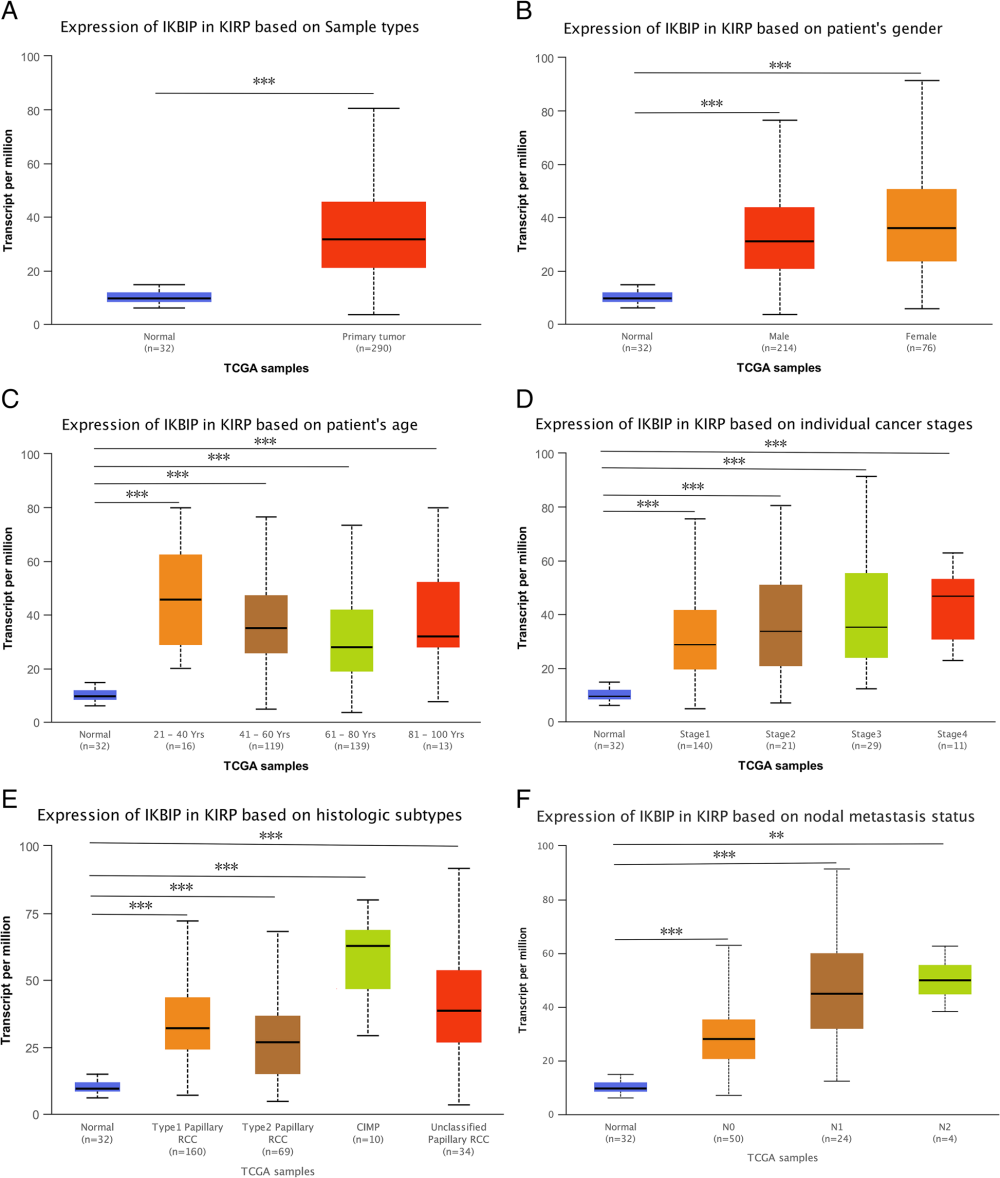
IKBIP transcription in subgroups of patients with PRCC, respectively based on gender, age, stage and other criteria (UALCAN). **A** Boxplot showing relative expression of IKBIP in PRCC and normal samples. **B** Boxplot showing relative expression of IKBIP in normal individuals of either gender and male or female PRCC patients. **C** Boxplot showing relative expression of IKBIP in normal individuals of any age or in PRCC patients. **D** Boxplot showing relative expression of IKBIP in normal individuals or in PRCC patients with stage 1,2,3 or 4. **E** Boxplot showing relative expression of IKBIP in normal individuals or in PRCC patients with different types. **F** Boxplot showing relative expression of IKBIP in normal individuals or PRCC patients with lymphatic metastasis. (**, *P* < 0.01; ***, *P* < 0.001)

**Fig. 3:**
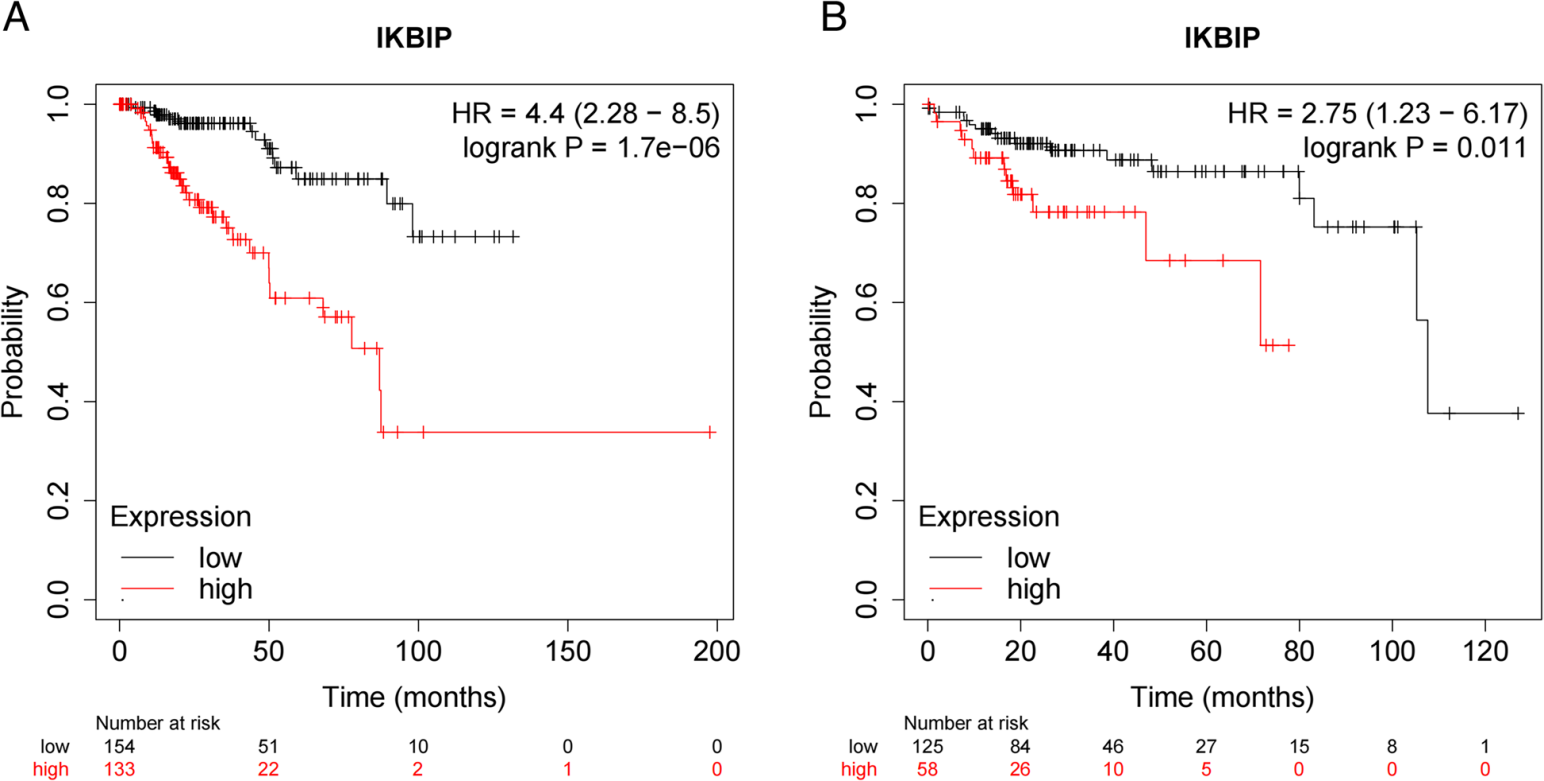
The survival outcome of IKBIP in PRCC patients (Kaplan–meier plotter). **A** Overall survival in TCGA KIRP cohort. **B** Disease-free survival in TCGA KIRP cohort

In this study, we analyzed the correlation between IKBIP mRNA expression and clinical-pathological features in PRCC patients by chi-square test and Fisher’s precision probability test. As shown in Table 1, high IKBIP mRNA expression was associated with age (*P* = 0.012), pathological stage (*P* = 0.013), pathological T stage (*P* = 0.006), pathological N stage (*P* = 0.005), respectively, but not with gender and pathological M stage (*P* > 0.05). And as summarized in Table 2, the univariate analysis showed that advanced stage, positive nodes, distant metastasis and higher IKBIP mRNA expression were significantly associated with worse overall survival of PRCC patients. Multivariate analysis using cox hazard proportional model revealed that IKBIP expression was an independent prognostic factor for patients of PRCC (*P* = 0.013). Furthermore, the expression profiles of IKBIP were significantly higher in PRCC patients than normal samples in sub-group analysis of multiple clinical-pathological features, including gender, age, tumor stages, histologic subtypes and nodal metastasis status (Fig. 2). And Kaplan–Meier analysis indicated that higher IKBIP mRNA expression was correlated with worse overall survival (*P* = 1.7E-6) (Fig. 3A) and disease free survival (*P* = 0.011) (Fig. 3B).

**Table 1:** Relationship between IKBIP expression and clinicopathological features of PRCC

Characteristic	levels	Low expression of IKBIP	High expression of IKBIP	p
**n**		144	145	
**Age, n (%)**	< = 60	55 (19.2%)	78 (27.3%)	0.012
	> 60	87 (30.4%)	66 (23.1%)	
**Gender, n (%)**	Female	33 (11.4%)	44 (15.2%)	0.195
	Male	111 (38.4%)	101 (34.9%)	
**Pathological stage, n (%)**	Stage I	97 (37.3%)	75 (28.8%)	0.013
	Stage II	10 (3.8%)	12 (4.6%)	
	Stage III	20 (7.7%)	31 (11.9%)	
	Stage IV	3 (1.2%)	12 (4.6%)	
**Pathological T stage, n (%)**	T1	109 (38%)	84 (29.3%)	0.006
	T2	12 (4.2%)	21 (7.3%)	
	T3	22 (7.7%)	37 (12.9%)	
	T4	0 (0%)	2 (0.7%)	
**Pathological N stage, n (%)**	N0	28 (36.4%)	21 (27.3%)	0.005
	N1	6 (7.8%)	18 (23.4%)	
	N2	0 (0%)	4 (5.2%)	
**Pathological M stage, n (%)**	M0	49 (47.1%)	46 (44.2%)	0.161
	M1	2 (1.9%)	7 (6.7%)	
**OS event, n (%)**	Alive	131 (45.3%)	114 (39.4%)	0.006
	Dead	13 (4.5%)	31 (10.7%)

**Table 2:** Univariate and Multivariate analysis of hazard factors of the prognosis of patients with PRCC

**Characteristics**	**Total(N)**	**Univariate analysis**		**Multivariate analysis**	
		**Hazard ratio (95% CI)**	***P* value**	**Hazard ratio (95% CI)**	***P* value**
**Age**	286				
< = 60	133	Reference			
> 60	153	0.944 (0.519–1.718)	0.851		
**Gender**	288				
Female	77	Reference			
Male	211	0.638 (0.331–1.230)	0.180		
**Pathological stage**	259				
Stage I	171	Reference			
Stage II	22	0.901 (0.201–4.040)	0.891	0.000 (0.000-Inf)	0.990
Stage III	51	4.827 (2.283–10.209)	** < 0.001**	1.950 (0.489–7.772)	0.344
Stage IV	15	16.276 (6.765–39.160)	** < 0.001**	88.777 (8.709–904.951)	** < 0.001**
**Pathological T stage**	286				
T1	192	Reference			
T2	33	2.983 (1.233–7.220)	**0.015**	1.215 (0.232–6.372)	0.818
T3	59	7.395 (3.692–14.809)	** < 0.001**	1.000 (0.280–3.571)	1.000
T4	2	0.000 (0.000-Inf)	0.997	1.000 (1.000–1.000)	
**Pathological N stage**	77				
N0	49	Reference			
N1	24	4.822 (1.932–12.038)	** < 0.001**	1.398 (0.394–4.957)	0.604
N2	4	6.033 (1.548–23.508)	**0.010**	0.000 (0.000-Inf)	0.990
**Pathological M stage**	104				
M0	95	Reference			
M1	9	114.966 (22.481–587.925)	** < 0.001**	1.000 (0.098–10.194)	1.000
**IKBIP**	288				
Low	144	Reference			
High	144	3.703 (1.919–7.144)	** < 0.001**	5.071 (1.401–18.352)	**0.013**

### IKBIP co-expression networks in PRCC

**Fig. 4:**
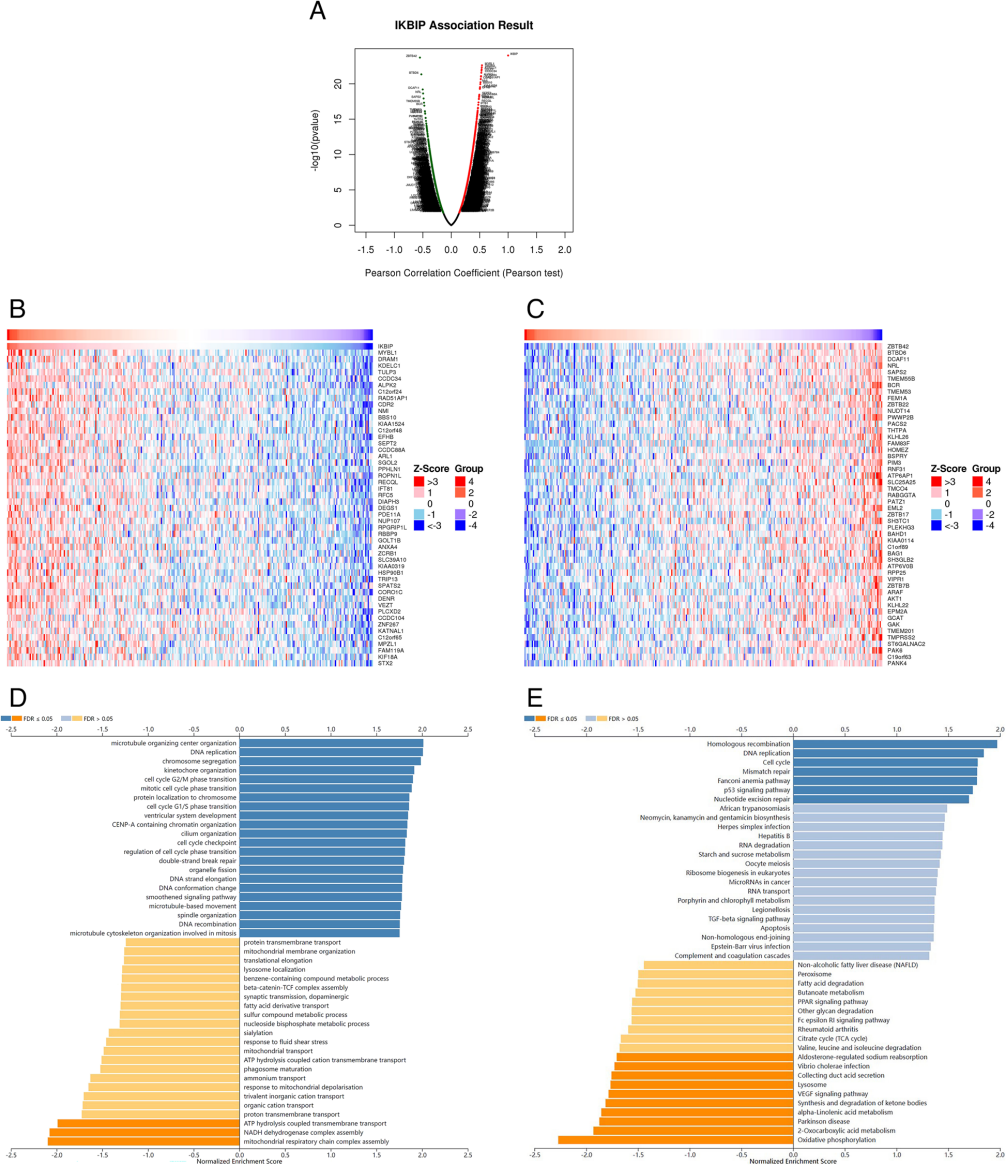
The co-expression genes with IKBIP in PRCC (LinkedOmics). **A** The significantly correlated genes with IKBIP identified by Pearson correlation in KIRP cohort. **B**, **C** Heatmaps showing top 50 genes positively and negtively correlated with IKBIP in KIRP. **D**, **E** GO annotations and KEGG pathways of IKBIP associated network in KIRP cohort

Based on LinkedOmics online database, we explored the biological significance in PRCC. In this database, 8433 genes were shown significant correlations with IKBIP (*P* < 0.05). As shown in Fig. 4A, 3141 genes were shown significant negative correlations with IKBIP in PRCC, whereas 5292 genes were shown positive correlations. Besides, the heat map displayed the top 50 significant genes which were positively and negatively correlated with IKBIP (Fig. 4B, C). MYBL1 (*r* = 0.540, *p* = 2.45E-23), DRAM1 (*r* = 0.537, *p* = 4.79E-23), KDELC1 (*r* = 0.534, *p* = 8.97E-23) and TULP3 (*r* = 0.532, *p* = 1.44E-22) showed a strong correlation with IKBIP mRNA expression in PRCC. Gene set enrichment analysis (GSEA) was used to show the significant gene ontology (GO) term annotation of IKBIP co-expressed genes. Then, we found that IKBIP co-expressed genes primarily participated in the regulation of the microtubule organizing center organization, DNA replication and chromosome segregation (Fig. 4D). Moreover, Kyoto Encyclopedia of Genes and Genomes (KEGG) [11–13] pathway analysis showed IKBIP co-expressed genes were enriched in homologous recombination, DNA replication, cell cycle, Mismatch repair, Fanconi anemia pathway, P53 signaling pathway and nucleotide excision repair. These results suggested that IKBIP might play an important role in PRCC by biological regulation (Fig. 4E).

### IKBIP protein expression is significantly up-regulated in human papillary renal cell carcinoma

**Fig. 5:**
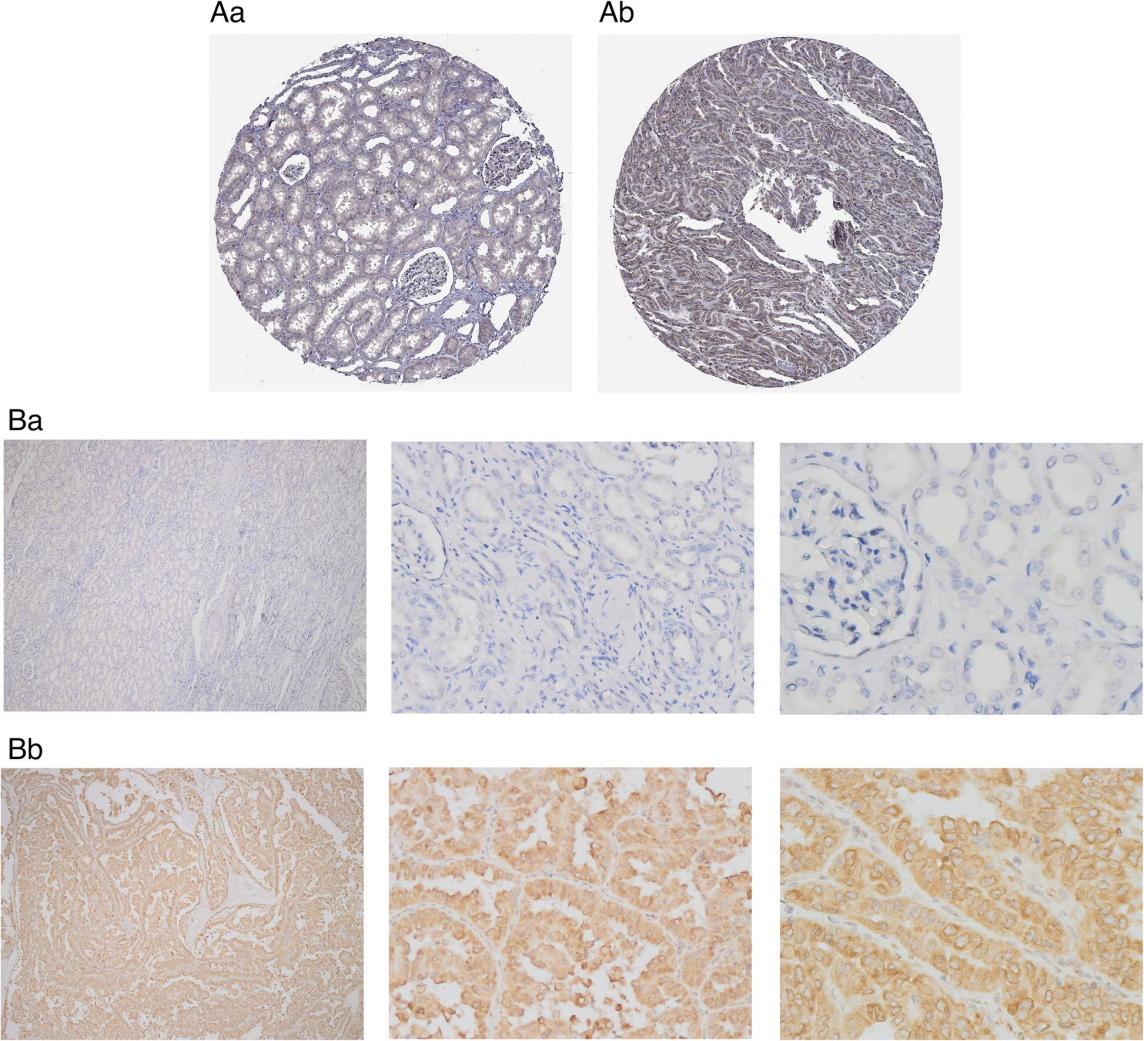
The protein expression level of IKBIP in PRCC and normal tissue. (A) The HPA : (a) normal kidney tissues (staining:not detected); (b) papillary renal cell carcinoma (staining:medium). (B) Immunohistochemical analysis: (a) normal kidney tissues (staining:not detected) ; (b) papillary renal cell

First, we retrieved the IHC pictures of PRCC and kidney normal tissues in the HPA database, and the IHC pictures displayed the positive staining of IKBIP expression, but negative or weakly staining in kidney normal tissues **(**Fig. 5A**)**. Then, we detected the protein expression of IKBIP in PRCC by Immunohistochemistry **(**Fig. 5B**).** Our result showed that IKBIP positive rate is 86.7% (13/15) in PRCC, whereas in the normal renal tissues, IKBIP positive rate is 33.3% (5/15), cytoplasmic IKBIP expression was stronger in primary cancer than normal renal tissues (*P* = 0.004). Thus, IKBIP may serve as a promising potential biomarker for PRCC diagnosis and prognosis.

## Discussion

Currently, few articles have reported the function of IKBIP in tumors, and previous studies have mainly focused on the role of IKBIP in glioma, but the specific mechanism is still unclear. In this study, we aimed to explore the role of IKBIP in the various tumors, especially in PRCC. Intriguingly, through integrative bioinformatics and Immunohistochemistry, we found the expression of IKBIP was higher in PRCC tissues than in normal renal tissues. Moreover, higher IKBIP expression was usually accompanied by a shorter overall or disease-free survival and more aggressive phenotype in PRCC. These results suggested that IKBIP played a crucial role in the malignant progression of PRCC. Understanding molecular mechanisms of IKBIP in PRCC may offer a new therapeutic target to improve prognosis for patients of PRCC.

In this study, we utilized GEPIA and TIMER database to explore the mRNA expression of IKBIP in multiple tumors, and we found that IKBIP mRNA levels were significantly higher in papillary renal cell carcinoma than in normal kidney tissue. Meanwhile, Immunohistochemistry results indicated that IKBIP protein expression levels were upregulated in papillary renal cell carcinoma. In addition, to probe the clinical correlation between IKBIP and PRCC, we evaluated the expression profiles of IKBIP in sub-group analysis of multiple clinical-pathological features, and we discovered that the expression of IKBIP was obviously higher in PRCC patients than in normal controls in subgroup analysis. Besides, high mRNA expression of IKBIP in PRCC was related to poor overall and disease-free survival. Thus, These results enlightened us that IKBIP might accelerate malignant progression of PRCC.

To explore the molecular mechanisms of IKBIP in PRCC. We applied KEGG pathway analysis based on the WebGestalt database to elucidate signaling pathway events in controlling abnormal IKBIP expression. It demonstrated that abnormal expression of IKBIP is involved in several pathways in PRCC, including homologous recombination, DNA replication, cell cycle, mismatch repair, Fanconi anemia pathway, P53 signaling pathway and nucleotide excision repair, these pathways may cause genomic instability.And From our findings, we can infer that these genes were apparently related to several cancer-associated signaling pathways. IKBIP might promote malignant progression of PRCC through these pathways. As reported by previous studies, IKBIP, regulated by P53, can induce apoptosis [6], in line with the results that IKBIP showed robust correlation with P53 signaling pathway presented by KEGG pathway analysis. But our findings revealed that IKBIP played a vital role in the malignant progression of PRCC. As a consequence, we considered that IKBIP might have dual influences on PRCC. And in PRCC, proapoptotic function of IKBIP was attenuated by other cancer-associated signaling pathways. As Yang etc. [8] reported, IKBIP might be a key molecule for EMT induction and the robust tumor-induced effect through EMT induction and immune inhibition overwhelmed the proapoptotic function. Certainly, in some malignancies, high proliferation is often accompanied by high apoptosis. Maybe we can explore whether there is a parallel relationship between proliferation and apoptosis in PRCC.

Cell cycle pathways are correlated with cellular proliferation [14]. Cell cycle progression,which is mainly driven by cyclin-dependent kinases, is the core event in all proliferating cells [15]. As Li etc. [16] reported, IKBIP promote cell proliferation and accelerate malignant progression of GBM by maintaining stability of CDK4. Combined with enrichment analysis of KEGG signaling pathway, We can also explore the relationship between IKBIP and cell cycle pathway in PRCC. Furthermore, an altered Fanconi anemia pathway causes genomic instability and has a predisposition to cancer [17], but more remains to be learned, especially in PRCC. In addition, according to previous reports, Mismatch repair causes predisposition to cancer, such as colorectal cancer and endometrioid carcinoma. And Mismatch repair proteins, including MSH/MLH/PMS proteins, loss in some tumors give rise to hypermutability, a phenomenon also known as microsatellite instability [18]. As a result, we speculate that high expression of IKBIP in PRCC may cause genomic instability, thus increasing the risk of developing cancer. But there are still more specific mechanisms that need to be explored.

Overall, our results verified the prognostic value of IKBIP in PRCC. But there are still some limitations. We mainly conducted bioinformatics to explore the role of IKBIP in PRCC, and the total number of samples for Immunochemistry is too small. In addition, because of the restriction of our laboratory conditions, we can not complete the cell function experiment of IKBIP in renal papillary cell carcinoma cell lines. More experiments should be carried out to confirm our results.

## Conclusions

Collectively, this article provided multi-level evidence for the conclusion that IKBIP expression was associated with malignant progression of PRCC and could cause worse survival for patients. Furthermore, IKBIP might promote tumorigenesis through several cancer-associated signaling pathways. Our findings can deepen the understanding of molecular profiles of IKBIP in PRCC. And IKBIP might be a promising biomarker with great potential in the future.

## Data Availability

All data generated or analyzed during this study are included in this published article.
